# Mechanical Properties of Calcium Fluoride-Based Composite Materials

**DOI:** 10.1155/2016/2752506

**Published:** 2016-11-28

**Authors:** Monika Łukomska-Szymańska, Joanna Kleczewska, Joanna Nowak, Mariusz Pryliński, Agata Szczesio, Magdalena Podlewska, Jerzy Sokołowski, Barbara Łapińska

**Affiliations:** ^1^Department of General Dentistry, Medical University of Lodz, 215 Pomorska St., 92-213 Lodz, Poland; ^2^Laboratory of Material Studies, Medical University of Lodz, 215 Pomorska St., 92-213 Lodz, Poland; ^3^Department of Biomaterials and Experimental Dentistry, University of Medical Sciences, Poznan, 70 Bukowska St., 60-812 Poznan, Poland

## Abstract

Aim of the study was to evaluate mechanical properties of light-curing composite materials modified with the addition of calcium fluoride. The study used one experimental light-curing composite material (ECM) and one commercially available flowable light-curing composite material (FA) that were modified with 0.5–5.0 wt% anhydrous calcium fluoride. Morphology of the samples and uniformity of CaF_2_ distribution were analyzed using Scanning Electron Microscopy (SEM) and Energy Dispersive Spectroscopy (EDS). Mechanical properties were tested after 24-hour storage of specimens in dry or wet conditions. Stored dry ECM enriched with 0.5–1.0 wt% CaF_2_ showed higher tensile strength values, while water storage of all modified ECM specimens decreased their tensile strength. The highest Vickers hardness tested after dry storage was observed for 2.5 wt% CaF_2_ content in ECM. The addition of 2.0–5.0 wt% CaF_2_ to FA caused significant decrease in tensile strength after dry storage and overall tensile strength decrease of modified FA specimens after water storage. The content of 2.0 wt% CaF_2_ in FA resulted in the highest Vickers hardness tested after wet storage. Commercially available composite material (FA), unmodified with fluoride addition, demonstrated overall significantly higher mechanical properties.

## 1. Introduction

Although constant development in dental material science, there has been no reconstructive material found that would perfectly restore hard dental tissues. Among main features of ideal reconstructive material are the ability to create durable bonding with hard dental tissue, biocompatibility, proper physical and mechanical properties, and good esthetics. Composite material possesses most of these features, yet it undergoes various modifications of chemical composition that would enhance the clinical performance including secondary caries prevention. Nowadays, one of the main objective in dental materials science is the introduction into material's composition compounds with antibacterial activity such as chlorhexidine digluconate (CHG), chlorhexidine acetate (CHA), quaternary ammonium dimethacrylate (QADM), or amorphous calcium phosphate (ACP). Another trend is modification of restorative materials with fluoride compounds such as NaF, CaF_2_, SnF_2_, SrF_2_, KF, which would release fluoride ions and contribute to remineralization of dental tissue within the cavity and in the environment surrounding the restoration [1]. Cariostatic effect of fluoride ions is widely documented and clinically proven [2–4] and encourages researchers to develop novel fluoride-based restorative materials [5, 6]. In order to enhance fluoride ions activity, combination with calcium compounds has been introduced [7].

Kulshrestha et al. [8] proved that CaF_2_ nanoparticles (CaF_2_-NPs) show strong antibacterial activity against* S. mutans* resulting in almost 90% reduction of biofilm formation, reduced bacteria acid, and exopolysaccharides production. At low pH, fluoride and hydrogen ions bind creating hydrofluoric acid (HF). HF penetrates bacterial membrane, dissociates inside bacteria, and causes acidification of cytoplasm and enzymes inhibition (enolase and ATPase) [8–10]. Fluorides in very high concentrations (3040–5700 ppm) cause bacteria cell death [11]. Moreover, fluoride also adversely influences metabolism and adhesion of bacteria cells [6, 12–15]. In presence of calcium fluoride nanoparticles, microbes showed decreased adhesion to tooth surface and to biofilm and greater sensitivity to acidic environment. Additionally, CaF_2_ nanoparticles restrain biofilm formation and as a consequence they reduce caries lesions development, due to great fluoride ion release and its influence on bacteria.

Calcium, as the enamel building element, also induces remineralization. Introducing calcium fluoride to dental composite materials is fully justified with secondary caries prevention. Still, the influence of fluoride compounds on mechanical performance of modified materials is under discussion and needs further studies.

Given wide and positive effect of fluoride ions on dental tissues, it is important to optimize fluoride content in composite materials so as it would induce cariostatic properties, but without deterioration of material mechanical properties.

## 2. Aim of the Study

The purpose was to evaluate mechanical parameters of two light-curing composite materials modified with the addition of calcium fluoride.

## 3. Materials and Methods

The study used two light-curing composite materials and calcium fluoride. One of the tested materials was the experimental light-curing composite material (ECM) developed in Laboratory of Material Studies in Medical University of Lodz (Poland). The ECM is based on dimethacrylic resins: Bis-GMA and TEGDMA (Sigma-Aldrich, USA), 35 wt% of precipitated silica filler (Arsil, Z. Ch. Rudniki, Poland) modified with 3-methacryloxypropyltrimethoxysilane (A-174, Sigma-Aldrich, USA), and additions such as camphorquinone (CQ, Sigma-Aldrich, USA), dimethylaminoethyl methacrylate (DMAEMA, Sigma-Aldrich, USA) and 2,6-di-t-butyl-p-cresol (BHT, Sigma-Aldrich, USA). The other tested material was flowable light-curing composite material Flow-Art (FA) (Arkona, Poland, shade A2, Lot 2013-08-21, Lot 2013-08-20, Lot 2013-08-22), containing 64 wt% of fillers (Al-Ba-F-Si glass, Al-Na-Ca-Si glass, pyrogenic silica). Both materials were modified with calcium fluoride, 99%, pure, anhydrous (CaF_2_) (Acros Organics, Belgium, Lot: A0306774).

For both tested materials, 6 study groups were established depending on the amount of the CaF_2_ added to the ECM and Flow-Art material (Tables 1 and 2). For each tested material, specimens without CaF_2_ addition served as a control group.

All mixtures were made based on 5.00 g of flowable composite, Flow-Art or ECM. Small portions (0.020–0.025 g) of calcium fluoride were weighed on an analytical balance and carefully grinded with the base composite (FA or ECM) in an agate mortar, until the desired homogeneity has been achieved.

Specimens of modified materials were fabricated using silicone molds. For the tensile strength test, cylindrical molds (3.0 mm thick and 6.0 mm in diameter) were fabricated. Composite materials were applied in layers and polymerized for 20 seconds per layer with Megalux polymerizing lamp with soft-start mode (Mega-Physic Dental, Germany). For hardness testing, composite discs 2.0 mm thick and 8.0 mm in diameter were fabricated, using silicone molds, in layering technique as described above.

Afterwards, all specimens were controlled and excess of material was removed by means of polishing with 140- and 320-gritt SiC papers. For each test, 24 specimens of tested materials were prepared. Specimens were stored in distilled water (subgroup 1, 12 specimens) and in dry conditions (subgroup 2, 12 specimens) for 24 hours.

For SEM-EDS evaluation, disc-shaped samples (3.0 mm thick and 6.0 mm in diameter) were fabricated in layering technique as described above. After 24 hours all specimens were polished with 140- to 2400-gritt SiC papers and then polished with 6 *μ*, 3 *μ*, and 1 *μ* diamond pastes.

### 3.1. Tensile Strength

Tensile strength of materials was tested with diametral tensile strength test (DTS) in universal testing machine (Zwick Z020, Zwick/Röell, Germany), at crosshead speed of 0.5 mm/min. The applied force, in the plane of its application, caused tensile stress in the material. Maximum force [N], causing specimen fracture, was recorded by the computer. DTS [MPa] values were calculated by the formula:

(1) $$\begin{array}{c} DTS=\frac{2F}{\pi dh}, \end{array}$$

where *F* is maximum force applied [N], *d* is diameter of the specimen [mm], and *h* is height of the specimen [mm].

### 3.2. Hardness

Hardness was measured using the Vickers hardness test method. The method involves the Vickers indenter, 136° diamond pyramid-shaped, forced into the tested specimen with definite load application and measuring dimensions of the indentation afterwards. The values obtained by the test are in the units known as Vickers Hardness Numbers (VHN, kg/mm^2^).

In order to perform the Vickers hardness test, Indentec ZH*μ*-SH*μ* microhardness tester (Zwick/Röell, Germany) with automatic indentation measurement was used. The indenter was forced into tested specimens with the load of 1 kg for 10 seconds. The distance between the edge of each indentations impress was at least thrice as long as the diagonal of the mark left by the indenter [16]. Vickers hardness was calculated by the formula:

(2) $$\begin{array}{c} HV=1.8544\frac{F}{d^{2}}, \end{array}$$

where *F* is the applied load [N] and *d* is the average length of the diagonal left by the indenter [mm].

### 3.3. SEM-EDS Analysis

Microstructure and chemical composition specimens were observed using the scanning electron microscopy (SEM) (S-4700, 15 kV, Hitachi, USA) with elemental dispersion spectroscopy (EDS) detector (EDS Thermo NORAN, Thermo Fisher Scientific, USA). Specimens were coated with platinum-palladium alloy prior testing. Specimens were analyzed in high vacuum conditions. SEM images were taken from representative areas of each specimen at two different magnifications (×1000 and ×5000). EDS spectra and element maps were taken at ×5000 magnification.

### 3.4. Statistical Analysis

Measurable variables (numeric, interval) were described using the measures of position, the mean value (M), median (Me), lower quartile (Q_1_), upper quartile (Q_3_), and interquartile range (IQR); measures of dispersion, the standard deviation (SD), standard error of mean (SE), 95% confidence interval (95% CI), and a trait's minimum and maximum values.

In the course of statistical analysis the following tests of significance were performed: the Shapiro-Wilk test for normality; Levene's test for the homogeneity of variances; a one-way and two-way analysis of variance (ANOVA) without replication; the Mann-Whitney ranks-sum *U*-test; the Kruskal-Wallis equality-of-populations rank test; generalized linear models.

A level of *p* < 0.05 was considered statistically significant. The statistical analysis of the study results was carried out using the Stata®/Special Edition, version 14.1 software package (StataCorp LP, College Station, Texas, USA).

## 4. Results

### 4.1. Diametral Tensile Strength

Diametral tensile strength test results for ECM and FA specimens stored in air and distilled water are shown in Table 3.

Among ECM specimens stored in dry conditions, DTS values in group ECM 0.5 and ECM 1.0 were statistically higher than in the other tested groups, while for subgroup stored in water, the highest DTS values of all tested groups were observed in control group.

For FA specimens stored in dry conditions, only group FA 1.0 showed significantly higher DTS values than the control group, while, for groups FA 2.0, FA 2.5, and FA 5.0, DTS values were statistically lower than for other test groups. Among FA specimens stored in water, the control group showed statistically higher DTS values than the other tested groups.

### 4.2. Hardness

Vickers hardness test results for ECM and FA specimens stored in dry conditions and in distilled water are shown in Table 4.

For ECM specimens stored in dry conditions, hardness values in group ECM 2.5 were statistically higher than in other tested groups. For ECM specimens stored in water, hardness values in groups ECM 0.5 and ECM 1.5 was statistically higher than in other tested groups.

Among FA specimens stored in dry conditions, only hardness values in group FA 1.0 were statistically higher than in other tested groups. On the other hand, for specimens stored in water, group FA 2.0 showed higher hardness than other tested groups.

### 4.3. SEM-EDS Analysis

**Figure 1:**
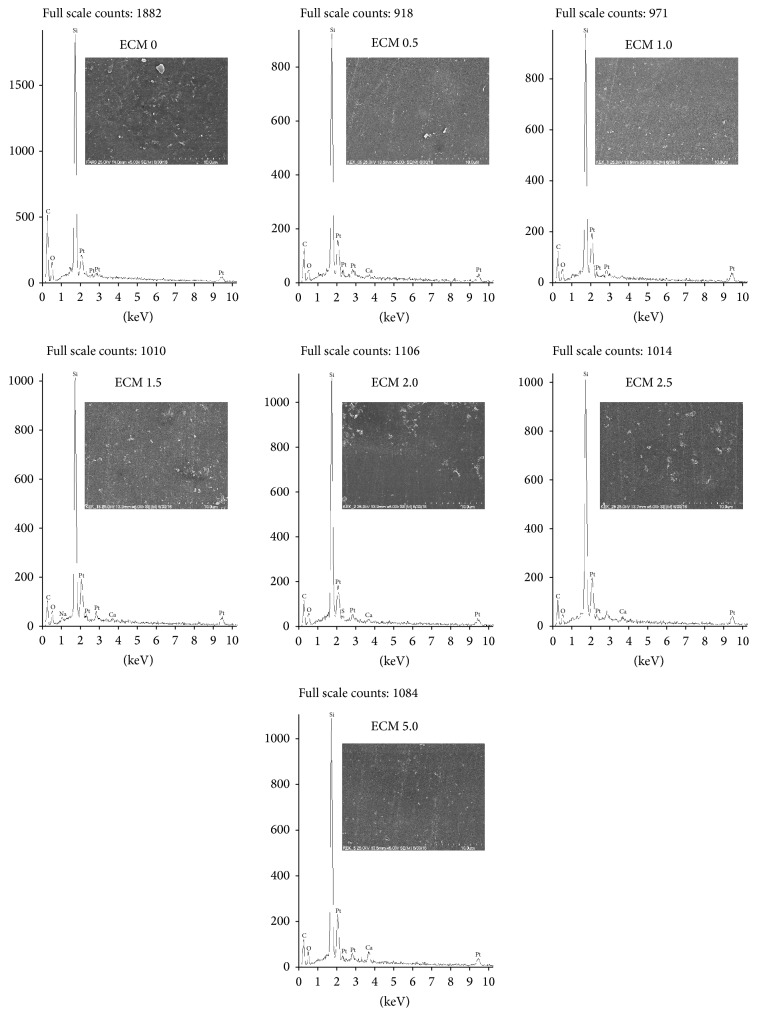
EDS spectra together with exemplary SEM images of experimental composite material (ECM) specimens: ECM 0 (control), ECM 0.5, ECM 1.0, ECM 1.5, ECM 2.0, ECM 2.5, and ECM 5.0, at ×5000 magnification.

SEM-EDS analysis of composites studied was conducted to visualize the morphology of the materials and the distribution of CaF_2_ in the sample volume. Significant differences in the internal structure between the ECM and FA were observed. Problems in obtaining satisfactory SEM images of good quality for ECM specimens are noticeable (Figure 1). Difficulties encountered are probably connected with the specific ECM composition, that is, very low filler loading (only 35 wt.%) and its fine sizes. However some silica agglomerates can be observed at the images. The intensity of calcium ions peak on EDS spectra for ECM samples changes with the increase of calcium fluoride content and reaches the highest values for the ECM modified with 5.0 wt% of CaF_2_.

**Figure 2:**
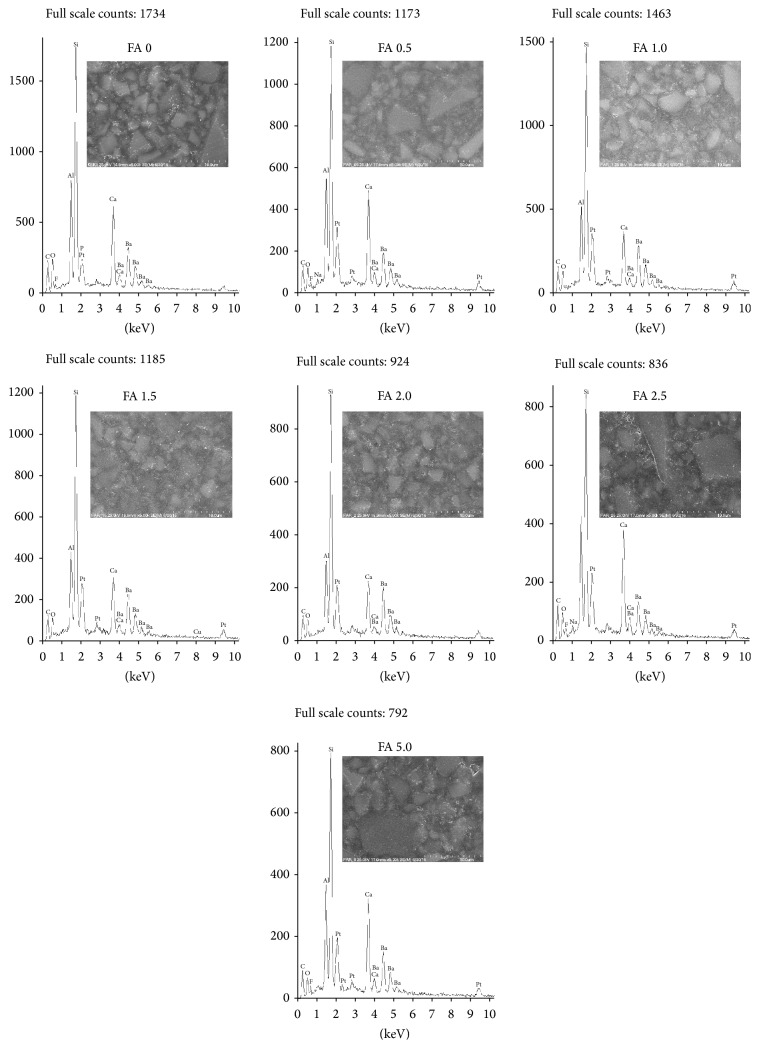
EDS spectra together with exemplary SEM images of flow-art (FA) specimens: FA 0 (control), FA 0.5, FA 1.0, FA 1.5, FA 2.0, FA 2.5, and FA 5.0, at ×5000 magnification.

All of the FA specimens (Figure 2) showed uniformly distributed glass filler particles of various sizes throughout the entire samples volume. No significant changes in the microstructure of materials after modification with CaF_2_ were observed. The differences between the calcium content for the Flow-Art material modified with calcium fluoride were very hard to notice on EDS spectra due to calcium content in one of the glass filler in FA composite.

## 5. Discussion

Modification of composite materials by introduction fluoride compounds seems to be very promising field of research. Those compounds show proven antibacterial and cariostatic activity [2–4]. It is expected that composite materials modified with fluoride compounds like calcium fluoride would also demonstrate antibacterial and cariostatic activity. Another very important clinical issue is mechanical properties of such fluoride-enriched composite materials. In the current study, two resin-based composite materials have been tested: commercially available and experimental one.

The commercially available composite material, as delivered by the manufacturer, demonstrated overall significantly higher mechanical properties (DTS, HV) than experimental one. Flow-Art showed diametral tensile strength above 30 MPa, while for experimental material the value ranged from 18 to 30 MPa. Average DTS values for common composite materials exceed 30 MPa [17]. Therefore, experimental composite material tested in the study would not be applicable in areas of substantial occlusal loading.

Flow-Art material showed hardness at HV 45–50, while ECM's hardness drops below HV 30. The differences between hardness values for various CaF_2_ content are not significant and may indicate that calcium fluoride addition does not change the polymerization conditions and does not interfere the curing process. Composite materials used in dentistry should demonstrate minimum hardness at level of HV 40–50 [18]. The aim of a restorative material should be to perfectly mimic the tissue that it needs to substitute, namely, enamel and dentine. The average hardness values of dental tissues range from 250 to 360 VHN for enamel and from 50 to 70 KHN for dentin [19]. However, these values show significant variations, although in dentin they are less pronounced. Craig and Peyton [20] reported that enamel hardness ranges from 344 ± 49 to 418 ± 60 VHN; Collys et al. [21] reported that enamel hardness ranges from 369 ± 25 to 431 ± 35; and Wilson and Love [22] reported that enamel hardness ranges from 263 ± 26 to 327 ± 40. The microhardness of the occlusal enamel varied from 359 to 424 VHN and that of the cervical enamel from 227 to 342 VHN [21]. Variations in the hardness values may result from histology features, chemical composition of dental tissues, and specimen preparation and load or reading error in indentation length (IL). Most of the conventional dental composites achieve top surface hardness of HV 70–110 [23, 24]. Given good results of hardness test, the commercial material (FA) could be used as universal restorative composite material. But for experimental composite material, low hardness narrows its clinical application to lining material or as class V and deciduous teeth restorative material.

The hardness of resin-based materials highly depends on the amount and hardness of filler particles; therefore composite materials with high filler content demonstrate favorable resistance to occlusal loads [25]. The results presented in the current study also confirm those findings. Material of lower hardness, ECM, contained less inorganic filler in comparison to material that exhibits higher hardness values (Flow-Art).

Considering the influence of calcium fluoride content on Flow-Art performance, it has been noted that the best mechanical properties of the modified material were obtained at 0.5% CaF_2_ content, after both dry and wet storage. Such CaF_2_ percentage in addition to material composition did not deteriorate tested mechanical parameters, allowing for clinical acceptance. In case of the experimental material, 1.0% calcium fluoride content was found optimal in dry storage conditions and both 0.5% and 1.0% CaF_2_ content, after water storage. In each group, material's mechanical properties remained similar to those of unmodified experimental material. Still, mechanical resistance of modified and unmodified ECM was inadequate for occlusal loading. Such material cannot serve as an universal restorative material.

Given difficulties with homogenous dispersion of inorganic additives in highly viscous organic matrix, the fillers amount in experimental composite material (ECM) was deliberately lowered down to 35 wt%. Thereby, composite material of low viscosity obtained served only as the base for introduction of calcium fluoride. It seems that any change in mechanical properties of ECM (with low filler content), caused by introduction of relatively small amount of CaF_2_, would be easier to detect. On the other hand, as for Flow-Art composite material, with relatively high filler content, small amount of additives would not change high performance of the material or these changes would be difficult to detect. Study results seem to confirm that hypothesis: changes in mechanical properties of FA composite are present at 2.0 or higher wt% CaF_2_ addition. DTS values of FA dropped significantly when CaF_2_ percentage content was high (5.0 wt%). It is suspected that decrease in mechanical properties of FA is due to the collapse and disintegration of matrix-fillers system. Yet, no changes in microhardness observed may indicate, although only indirectly, that calcium fluoride addition did not violate/disrupt polymerization of Flow-Art composite.

Similar research was conducted by Xu et al. [26]. They evaluated mechanical properties and fluoride ion release from experimental composite material modified with calcium fluoride nanoparticles incorporated into polymer matrix. The modified experimental composite containing 10 wt% or 20 wt% CaF_2_ showed higher TFS values and elasticity modulus when compared to two commercial materials (Vitremer, Heliomolar). Relatively high fluoride ion release values combined with relatively low fluoride content in filler were explained with small size of filler nanoparticles (1.0 *μ*m).

Dental materials used for restoration of hard dental tissues must present stable mechanical properties in moist oral cavity environment. In the study, the comparison of the materials' mechanical properties in both dry and wet conditions has been made. The hardness of both tested materials was significantly lower after water storage, while DTS values showed no such relation. Only ECM and ECM modified with 5.0 wt% CaF_2_ presented significantly higher tensile strength after dry storage.

Deterioration of materials mechanical properties after water storage, when compared to dry storage, is probably caused by hydrolytic degradation. Water induces loosening of polymeric network. While cross-linked dimetacrylic resins is swelled with water, ester bonds undergo hydrolysis that causes network weakening and degradation. Moreover, in wet conditions, previously unreacted monomers and oligomers may be released to the external environment, water. Even though CaF_2_ is highly insoluble in water, it may be leached from composite weakened matrix to water environment. That may lead to the increase in microporosity of composite surface and deterioration of its mechanical properties such as DTS and hardness. The effect was observed in the study and was more prominent in case of ECM due to its high resin content responsible for water sorption.

Hydrolytic degradation of composite materials is present mainly in organic matrix and at matrix-filler interface. The rate and susceptibility of composite material to hydrolytic degradation depend on percentage content of resins and their bond quality to filler particles. Considering the higher resin content in experimental composite material than in commercial one (Flow-Art), the former is assumed to be more susceptible to hydrolytic degradation.

Study results show that mechanical properties of fluoride-based composite materials depend on the source, not the amount of fluoride compound added, and may be maintained by proper size of additives particle and filler content/volume. The present study indicated that introduction of 0.5–1.0 wt% soluble fluoride salt into tested materials did not induce negative effect on their mechanical-physical properties. However, due to high occlusal stresses in oral cavity, those materials cannot be used in restoration of all class cavities. Indication for use of such fluoride-based materials could include class V cavities in permanent teeth as well as all classes of cavities in deciduous teeth. Moreover, fluoride-based flowable composite materials may serve as base liner that are not subjected to high occlusal forces. In deep cavities, using the material with remineralising and bacteriostatic effect seems to be clinically justified.

## 6. Conclusions

The best mechanical properties of flowable composite materials modified with CaF_2_ were obtained when 0.5 wt% CaF_2_ was added to commercial composite and 1.0 wt% to experimental composite. The hardness of materials tested after dry storage was higher than after water storage. Commercially available composite material showed higher mechanical properties than the experimental one. Further studies on fluoride-based composite materials should be conducted including ion release as well as microbiological properties evaluation.

## Figures and Tables

**Table 1:** Specimens of experimental light-curing composite material (ECM).

ECM 0	ECM 0.5	ECM 1.0	ECM 1.5	ECM 2.0	ECM 2.5	ECM 5.0
ECM control group	ECM + 0.5 wt% CaF_2_	ECM + 1.0 wt% CaF_2_	ECM + 1.5 wt% CaF_2_	ECM + 2.0 wt% CaF_2_	ECM + 2.5 wt% CaF_2_	ECM + 5.0 wt% CaF_2_

**Table 2:** Specimens of Flow-Art composite material (FA).

FA 0	FA 0.5	FA 1.0	FA 1.5	FA 2.0	FA 2.5	FA 5.0
Flow-Art control group	Flow-Art + 0.5 wt% CaF_2_	Flow-Art + 1.0 wt% CaF_2_	Flow-Art + 1.5 wt% CaF_2_	Flow-Art + 2.0 wt% CaF_2_	Flow-Art + 2.5 wt% CaF_2_	Flow-Art + 5.0 wt% CaF_2_

**Table 3:** DTS test results for ECM and FA specimens.

Group	CaF_2_ content	Storage conditions			
		Dry (subgroup 1)		Distilled water (subgroup 2)	
		Mean [MPa]	Standard deviation (SD)	Mean [MPa]	Standard deviation (SD)
ECM 0	0.0%	18.62	6.54	31.55	7.91
ECM 0.5	0.5%	25.51	4.99	21.45	4.61
ECM 1.0	1.0%	26.96	3.51	27.24	2.10
ECM 1.5	1.5%	19.07	4.67	16.65	4.24
ECM 2.0	2.0%	21.68	5.50	15.84	5.53
ECM 2.5	2.5%	16.63	5.18	19.94	7.26
ECM 5.0	5.0%	17.10	8.39	26.86	7.85

FA 0	0.0%	28.07	6.04	31.70	8.56
FA 0.5	0.5%	26.47	2.13	25.94	3.66
FA 1.0	1.0%	27.58	4.13	27.74	3.22
FA 1.5	1.5%	30.65	5.85	29.68	5.37
FA 2.0	2.0%	23.85	4.24	23.63	5.38
FA 2.5	2.5%	22.55	3.67	24.19	3.51
FA 5.0	5.0%	21.40	6.64	22.59	4.00

**Table 4:** Vickers hardness test results for ECM and FA specimens.

Group	CaF_2_ content	Storage conditions			
		Dry (subgroup 1)		Distilled water (subgroup 2)	
		Mean [VHN]	Standard deviation (SD)	Mean [VHN]	Standard deviation (SD)
ECM 0	0.0%	23.73	1.31	17.67	4.90
ECM 0.5	0.5%	16.20	4.65	21.67	6.19
ECM 1.0	1.0%	23.33	1.52	19.56	1.19
ECM 1.5	1.5%	25.44	6.02	19.86	6.89
ECM 2.0	2.0%	25.33	6.88	15.78	2.44
ECM 2.5	2.5%	29.40	6.16	13.33	2.50
ECM 5.0	5.0%	24.90	5.71	11.83	1.48

FA 0	0.0%	50.11	7.05	45.00	0.00
FA 0.5	0.5%	49.25	2.16	43.82	2.11
FA 1.0	1.0%	52.40	0.93	46.30	1.95
FA 1.5	1.5%	50.43	1.92	40.13	7.68
FA 2.0	2.0%	44.59	4.98	49.11	5.69
FA 2.5	2.5%	47.29	6.50	41.08	1.57
FA 5.0	5.0%	50.00	4.07	44.00	2.09
